# Simultaneous versus staged major hepatectomy (≥3 liver segments) for outcomes of synchronous colorectal liver metastases: A systematic review and meta‐analysis

**DOI:** 10.1002/cnr2.1617

**Published:** 2022-06-26

**Authors:** Jianwei Liu, Yong Xia, Xiaorong Pan, Zhenlin Yan, Lei Zhang, Zhao Yang, Yeye Wu, Hui Xue, Shilei Bai, Feng Shen, Kui Wang

**Affiliations:** ^1^ Department of Hepatic Surgery II Eastern Hepatobiliary Surgery Hospital, Navy Medical University Shanghai China; ^2^ Department of Hepatic Surgery IV Eastern Hepatobiliary Surgery Hospital, Navy Medical University Shanghai China; ^3^ Shanghai Baoshan District Songnan Town Community Health Center Shanghai China

**Keywords:** colorectal, complications, liver metastases, mortality, prognosis, simultaneous hepatectomy, staged hepatectomy

## Abstract

**Background:**

Hepatectomy is an effective treatment for synchronous colorectal liver metastases (SCLM) patients. However, whether to choose simultaneous hepatectomy (SIH) or staged hepatectomy (STH) is still controversial, especially during major hepatectomy (≥3 liver segments).

**Aims:**

Compare the difference between the SCLM patients underwent SIH and STH, especially during major hepatectomy (≥3 liver segments).

**Methods and Results:**

A meta‐analysis was conducted by analyzing the published data on the outcomes of SCLM patients underwent SIH or STH from January 2010 to December 2020 from the electronic databases. A random‐effects model was used to derive pooled estimates of odds ratio (OR) with 95% confidence interval (CI) for the explored outcomes. Eventually, 18 studies, including 5101 patients, were included this study. The result of meta‐analysis showed that SIH did not increase postoperative complications (pooled OR: 1.037; 95% CI: 0.897–1.200), perioperative mortality (pooled OR: 0.942; 95% CI: 0.552–1.607), 3‐year mortality (pooled OR: 1.090; 95% CI: 0.903–1.316) or 5‐year mortality (pooled OR: 1.077; 95% CI: 0.926–1.253), as compared with STH. Subgroup analysis showed that, simultaneous major hepatectomy (SIMH) also did not increase postoperative complications (pooled OR: 0.863; 95% CI: 0.627–1.188) or perioperative mortality (pooled OR: 0.689; 95% CI: 0.290–1.637) as compared with staged major hepatectomy (STMH).

**Conclusion:**

Postoperative complications, perioperative mortality and long‐term prognosis had no significant difference between SIH and STH for SCLM patients. Besides, postoperative complications and perioperative mortality also had no significant difference between SIMH and STMH.

## 1 INTRODUCTION

Colorectal cancer is the most common malignant tumor in the world, which seriously threatens human health. According to the latest global tumor statistics, colorectal cancer is the fourth most common cancer and the second leading cause of cancer related death in the world.^1^ About 15%–25% of colorectal cancer is accompanied with synchronous colorectal liver metastases (SCLM),^2^, ^3^, ^4^, ^5^ and only one quarter of them are eligible for surgical resection.^6^ The simultaneous hepatectomy (SIH) and staged hepatectomy (STH) are effective surgical methods.^7^, ^8^ According to articles, the 5‐year overall survival (OS) rate of surgical treatment for SCLM can reach more than 50%.^9^, ^10^ However, the timing of surgery remains controversial.^11^, ^12^, ^13^, ^14^, ^15^, ^16^, ^17^, ^18^, ^19^ Some studies suggested that SIH could increase the risk of postoperative complications and perioperative mortality,^11^, ^12^, ^13^ while other studies did not support this conclusion.^14^, ^15^, ^16^ In addition, the difference in long‐term survival is also unclear between the SIH and STH.^17^, ^18^, ^19^

By reviewing the previous articles, we found that major hepatectomy (≥3 liver segments) is rarely reported in SIH for SCLM before 2010. However, articles on major hepatectomy in SIH have significantly increased in the past decade.^10^, ^11^, ^12^, ^13^, ^14^, ^15^, ^20^, ^21^, ^22^, ^23^, ^24^, ^25^, ^26^ Whether more patients received major hepatectomy can lead to differences in postoperative complications and perioperative mortality between simultaneous major hepatectomy (SIMH) and staged major hepatectomy (STMH) is unclear. And no meta‐analysis has been performed to investigate this issue.

In this study, we reviewed large number of articles published after 2010 to compare the difference in postoperative complications, perioperative mortality and long‐term prognosis between SIH and STH. Besides, stratified meta‐analyses were performed to compare postoperative complications and perioperative mortality between SIMH and STMH.

## 2 MATERIALS AND METHODS

### 2.1 Search strategy

**FIGURE 1:**
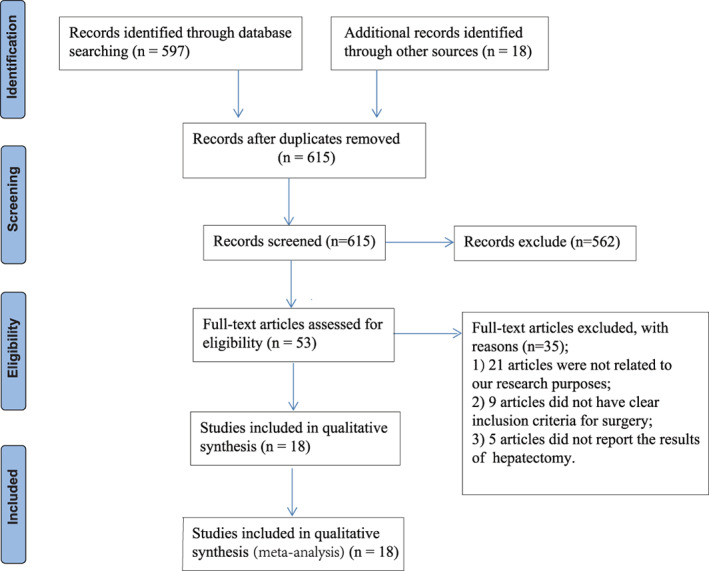
Flow chart of this study

Relevant articles published from January 2010 to December 2020 was searched by Medline, Embase, Ovid and Cochrane. The search terms included “colorectal cancer”, “liver metastases”, “simultaneous resection”, “staged resections”, “delayed resections” and “liver surgery”. All relevant titles, abstracts, conference and so on were evaluated independently by two investigators to determine whether they meet our research objectives and requirements. Then the full‐text of related articles were carefully reviewed and independent quality assessment was done by the two investigators. A third scholar would be consulted and make the decision when disagreement occured. Figure 1 showed the flow‐diagram of this study. And the study design conformed to the PRISMA guideline.^27^

### 2.2 Study selection

To ensure the quality of our study, only studies with complete articles were included, and abstracts, case reports and reviews were excluded.

The inclusion criteria were: (1) English articles published from January 2010 to December 2020; (2) the patients were diagnosed with SCLM at the first diagnosis; (3) SCLM was confirmed by pathology; (4) SCLM patients underwent SIH or STH in the same study; (5) There was at least one clearly reported including postoperative 3‐year OS, postoperative 5‐year OS, postoperative complications or perioperative mortality. Studies that did not meet the inclusion criteria were excluded.

### 2.3 Data extraction

After downloaded the full texts of related articles, data regarding the following aspects were extracted and recorded: authors, countries or regions, population, type of study (prospective or retrospective), number of patients underwent SIH and STH; sex and age of patients, location of the primary tumor (colon or rectum), number of transfusions, proportion of patients underwent major hepatectomy (≥3 liver segments), postoperative complications, perioperative mortality, 3‐year, 5‐year OS and the corresponding mortality. Ethics committee approval not received for this study as there are no human or animal subjects directly recruited.

### 2.4 Quality assessment

Two researchers independently evaluated the quality of articles according to the quality in prognosis studies (QUIPS) tool.^28^, ^29^ The authenticity and bias were evaluated through six aspects: participation; attrition; prognostic factor measurement; confounding measurement and account; outcome measurement; analysis and reporting.

### 2.5 Outcomes

Surgical safety which included postoperative complications and perioperative mortality was the primary focus of this study. Long‐term survival which included 3‐year and 5‐year mortality was the secondary focus. Postoperative complications or perioperative mortality were defined as adverse events or death within 90 days after surgery, and complications were classified according to Clavien classification.^30^ Stratified meta‐analyses was performed on patients who underwent SIMH and STMH.

### 2.6 Statistical analysis

Stata 12.0 software (Corp. STATA, Station college, TX) was used for data analysis. The pooled estimates of odds ratio (OR) and 95% confidence interval (CI) were obtained by random‐effects model. Chi‐squared test was used to analyze the heterogeneity and *I* ^2^ was used to analyze the degree of data inconsistency. A value of *p* < .05 was considered as significant difference and *I* ^2^ > 50% was considered as significant heterogeneity.^31^ In addition, sensitivity analyses were performed to investigate potential sources of bias in the results.

## 3 RESULTS

### 3.1 Literature search

Figure 1 shows the complete selection process of this study. A total of 615 studies were obtained after the preliminary search in Medline, Embase and other electronic databases using the keywords. Five hundred and sixty‐two articles were excluded after the initial screening and review of the titles and abstracts. The full‐texts of the remaining 53 articles were downloaded and re‐evaluated, and 35 studies were excluded due to irrelevance to the research purpose (*n* = 21), failure to clarify inclusion criteria for patients (*n* = 9), and failure to report the results of surgery including postoperative complications, perioperative mortality and postoperative OS (*n* = 5). The remaining 18 studies that included 5101 patients underwent SIH or STH were included and further analyzed.^10^, ^11^, ^12^, ^13^, ^14^, ^15^, ^16^, ^17^, ^18^, ^19^, ^20^, ^21^, ^22^, ^23^, ^24^, ^25^, ^26^, ^32^

### 3.2 Characteristics of the included studies

Of the 18 articles included in this study, 17 were retrospective studies,^10^, ^11^, ^12^, ^13^, ^14^, ^15^, ^16^, ^17^, ^18^, ^19^, ^20^, ^21^, ^22^, ^23^, ^24^, ^25^, ^32^ and one was prospective study.^26^ The geographical distribution of the included studies was America (*n* = 4),^10^, ^14^, ^15^, ^26^ China (*n* = 3),^2^, ^11^, ^19^ Canada (*n* = 2),^12^, ^24^ Japan (*n* = 2),^17^, ^21^ Korea (*n* = 2),^18^, ^32^ Romania (*n* = 2),^23^, ^25^ Italy (*n* = 1),^13^ England (*n* = 1)^22^ and India (*n* = 1).^16^ For the 5101 patients, 2365 (46.4%) patients underwent SIH and 2736 (53.6%) patients underwent STH. Of the 18 articles, 15 articles reported postoperative complications,^10^, ^11^, ^12^, ^13^, ^14^, ^15^, ^16^, ^17^, ^18^, ^19^, ^20^, ^21^, ^22^, ^23^, ^25^ 14 articles reported perioperative mortality^10^, ^11^, ^12^, ^13^, ^14^, ^15^, ^16^, ^17^, ^19^, ^20^, ^21^, ^22^, ^23^, ^25^and 16 articles reported long‐term survival after SIH or STH^10^, ^11^, ^12^, ^13^, ^15^, ^16^, ^17^, ^18^, ^19^, ^21^, ^22^, ^23^, ^24^, ^25^, ^26^, ^32^ (Tables 1 and 2).

**TABLE 1:** Main characteristics of literatures included in this systematic review and meta‐analysis

Author& time	*n* (SR/DR)	Male, %		Age		Colon/rectum	Transfusion (%)		Hepatectomy (≥3 segments)(%)		Study design	Area	Ref
		SR	DR	SR	DR		SR	DR	SR	DR			
Luo Y, 2010	405 (129/276)	58.9	56.5	58 (42–69)	60 (43–70)	199/206	NR	NR	56.8	57.1	Re	China	20
Kaibori M, 2010	74 (32/42)	64.3	53.1	65.0 ± 9.9	62.3 ± 9.3	55/19	11.0	15.0	NR	NR	Re	Japan	17
Brouquet A, 2010	115 (43/72)	53.5	61.1	56 (25–81)	58 (31–77)	62/53	16.0	13.0	35.0	66.0	Re	American	10
de Haas RJ, 2010	228 (55/173)	50.9	61.8	56.0 ± 12.0	58.0 ± 11.0	43/140	NR	NR	NR	NR	Re	India	16
Moug SJ, 2010	64 (32/32)	56.3	65.6	59 (53–79)	67 (37–82)	NR	NR	NR	21.9	21.9	Re	English	22
Abbott DE, 2012	144 (60/84)	66.7	58.3	58 (46–64)	53(46–61)	57/87	0	13.1	33.3	75.0	Re	American	14
Alexandrescu S, 2012	142 (117/25)	45.3	36.0	59.0 ± 10.8	56.7 ± 11.6	103/39	NR	NR	18.0	36.0	Re	Romania	23
Mayo SC, 2013	976 (329/647)	56.2	60.7	60.0 ± 30.1	61.0 ± 17.8	713/261	NR	NR	23.7	35.7	Re	American	15
Patrono D, 2014	106 (46/60)	52.2	61.2	63.6 ± 11.5	60.9 ± 9.1	85/21	65.2	60.0	28.3	46.7	Re	Italy	13
Fukami Y, 2015	63 (41/22)	43.9	54.5	65 ± 9	65 ± 7	35/28	NR	NR	22.0	31.8	Re	Japan	21
Yuan L, 2016	73 (60/13)	63.0	77.0	55.4 ± 11.6	54.5 ± 10.1	40/33	18.3	7.7	NR	NR	Re	China	19
Chan W, 2017	149 (96/53)	81.3	66.0	59.0 ± 9	59.0 ± 10.0	88/61	NR	NR	NR	NR	Re	Korea	32
Nanji S, 2017	226 (100/126)	54.0	56.0	61 (20–87)	62 (22–87)	NR	NR	NR	21.0	79.0	Re	Canadian	24
Alexandrescu S, 2017	300 (234/66)	56.4	53.0	61 ± 11	59 ± 9	207/93	NR	NR	18.9	30.3	Re	Romania	25
Silberhumer GR, 2017	429 (320/109)	53.1	62.4	58.6 ± 13.6	59.4 ± 12.3	233/196	NR	NR	33.4	72.5	Pr	American	26
Bogach J, 2019	1166 (442/724)	58.8	63.2	>60y:67.2	>60y:56.2	837/218	NR	NR	17.0	64.50	Re	Canada	12
Kye BH, 2019	208 (143/65)	66.2	71.3	>65y:35	>65y:21.5	108/100	NR	NR	NR	NR	Re	Korea	18
Wang LJ, 2020	233 (86/147)	52.3	67.3	57(33–82)	59 (32–77)	147/86	NR	NR	33.7	34.4	Re	China	11

Abbreviations: DR, delayed resection; *n*, number; *n*, number of included population in study; NR, no report; Pr, prospective; Re, retrospective; Ref, references; SR, synchronous resection.

**TABLE 2:** 3‐, 5‐year OS and mortality, corresponding complications and perioperative mortality of literatures included in this systematic review and meta‐analysis

Author & time	*n*	3‐years OS, %		3‐years mortality, *n*		5‐years OS, %		5‐years mortality, *n*		Complications, *n*		perioperative Mortality, *n*		Ref
		SR	DR	SR	DR	SR	DR	SR	DR	SR	DR	SR	DR	
Luo Y, 2010	405	NR	NR	NR	NR	NR	NR	NR	NR	61	150	2	6	20
Kaibori M, 2010	74	NR	NR	NR	NR	14.0	25.0	18	17	12	6	0	0	17
Brouquet A, 2010	115	65.0	58.0	15	30	55.0	48.0	19	37	20	37	2	2	10
de Haas RJ, 2010	228	74.5	70.5	14	51	NR	NR	NR	NR	6	44	0	1	16
Moug SJ, 2010	64	NR	NR	NR	NR	21.0	24.0	25	24	11	19	0	0	22
Alexandrescu S, 2012	142	52.2	42.4	56	14	21.9	14.1	91	21	41	7	7	3	14
Abbott DE, 2012	144	NR	NR	NR	NR	NR	NR	NR	NR	23	34	2	1	23
Mayo SC, 2013	976	61.1	60.0	128	259	43.8	45.6	185	352	63	128	9	21	15
Patrono D, 2014	106	55.0	56.0	21	26	34.0	33.0	30	40	27	32	1	0	13
Fukami Y, 2015	63	65.6	66.8	14	7	56.0	0	18	22	9	6	0	0	21
Yuan L, 2016	73	53.0	10.0	28	12	23.0	0	46	13	14	6	0	0	19
Chan W, 2017	149	64.6	77.2	34	12	NR	NR	NR	NR	NR	NR	NR	NR	32
Nanji S, 2017	226	NR	NR	NR	NR	36.0	51.0	64	62	NR	NR	NR	NR	24
Alexandrescu S, 2017	300	51.3	49.6	114	33	30.0	22.5	164	51	96	21	9	2	25
Silberhumer GR, 2017	429	64.0	60.5	115	43	38.5	38.9	197	67	NR	NR	NR	NR	26
Bogach J, 2019	1166	56.0	73.0	194	195	37.0	55.0	278	326	124	167	27	7	12
Kye BH, 2019	208	69.4	85.0	44	10	50.0	76.5	72	15	26	12	NR	NR	18
Wang LJ, 2020	233	55.8	59.9	39	59	44.2	38.8	48	90	42	51	0	0	11

Abbreviations: DR, delayed resection; *n*, number of included population in study; NR, no report; OS, overall survival; Ref, references; SR, synchronous resection.

### 3.3 Quality of the included studies

Table S1 listed the quality score of each article. Six articles scored 6–7 points, which were considered low risk of bias. Seven articles scored 8–9 points, which were considered medium risk of bias. Five articles scored ≥10 points and considered high risk of bias.

### 3.4 Primary outcome: compare the surgical safety between SIH and STH

**FIGURE 2:**
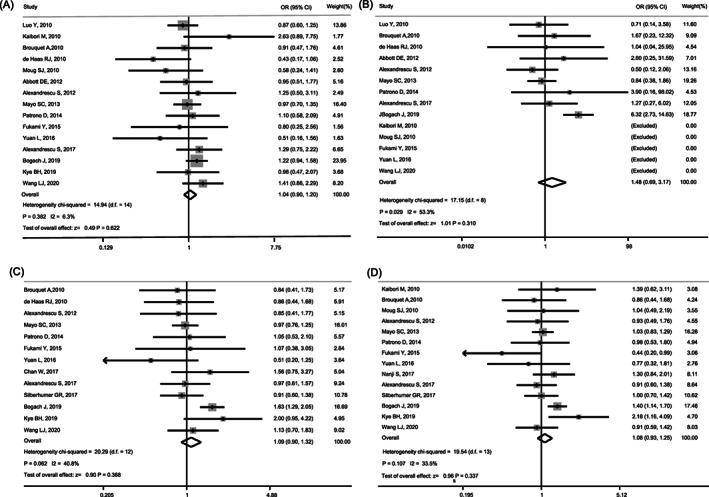
The impact of simultaneous hepatectomy (SIH) on surgical safety, long‐term prognosis compared with staged hepatectomy (STH). (A) The impact of SIH on postoperative complications compared with STH; (B) the impact of SIH on perioperative mortality compared with STH; (C) the impact of SIH on postoperative 3‐year mortality compared with STH; (D) the impact of SIH on postoperative 5‐year mortality compared with STH

Table 2 shows the postoperative complications and perioperative mortality. We performed meta‐analysis on 4307 patients for postoperative complications from 15 studies,^10^, ^11^, ^12^, ^13^, ^14^, ^15^, ^16^, ^17^, ^18^, ^19^, ^20^, ^21^, ^22^, ^23^, ^25^ and 4099 patients for perioperative mortality from 14 studies.^10^, ^11^, ^12^, ^13^, ^14^, ^15^, ^16^, ^17^, ^19^, ^20^, ^21^, ^22^, ^23^, ^25^ In 14 studies, five studies reported the perioperative mortality was zero for both SIH and STH.^11^, ^17^, ^19^, ^21^, ^22^ The complication rates were 575/1849 (31.1%) for SIH and 720/2458 (29.3%) for STH, and the corresponding perioperative mortalities were 59/1706 (3.5%) and 43/2393 (1.8%), respectively. The results of meta‐analysis showed that SIH did not increase the risk of postoperative complications (pooled OR: 1.037; 95%CI: 0.897–1.200; *p* = .622) or perioperative mortality (pooled OR: 1.482; 95% CI: 0.693–3.169; *p* = .310) as compared with STH (Figure 2A,B). In this analysis, there were mild and high heterogeneities for postoperative complications and perioperative mortality, respectively (*p* = .382; *I* ^2^ = 6.3%; *p* = .029; *I* ^2^ = 53.3%). The sensitivity analysis showed that there was little difference among the 15 studies for postoperative complications (Figure S1A). However, for perioperative mortality, there was one article that was significantly different as other articles,^12^ while the others were relatively similar (Figure S1B).

**FIGURE 3:**
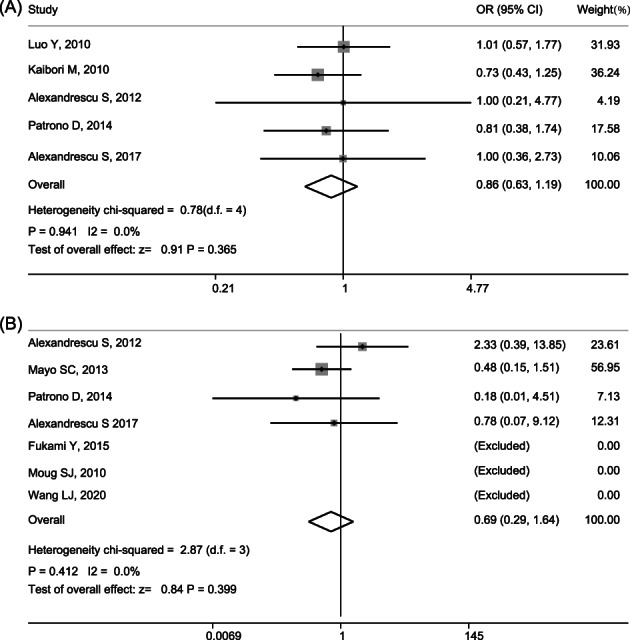
Stratified meta‐analysis according to patients underwent major hepatectomy (≥3 liver segments). (A) Subgroup of the impact of simultaneous hepatectomy (SIH) on postoperative complications compared with staged hepatectomy (STH); (B) subgroup of the impact of SIH on perioperative mortality compared with STH

The subgroup meta‐analysis for postoperative complication (five articles^13^, ^15^, ^20^, ^23^, ^25^) and perioperative mortality (seven articles^11^, ^13^, ^15^, ^21^, ^22^, ^23^, ^25^) was performed between SIMH and STMH. The postoperative complication rate was 91/194(46.9%) for SIMH and 180/457(39.4%) for STMH, and the perioperative mortality rate was 11/195 (5.6%) for SIMH and 12/390 (3.1%) for STMH. The results of subgroup meta‐analysis showed that, compared to STMH, SIMH did not increase the risk of postoperative complications (0.863; 95%CI: 0.627–1.188; *p* = .365) or perioperative mortality (pooled OR: 0.689; 95%CI: 0.290–1.637; *p* = .399) (Figure 3A,B). There was no heterogeneity in this analysis (*p* = .941; *I* ^2^ = 0; *p* = .412; *I* ^2^ = 0).

### 3.5 Secondary outcome: compare the long‐term survival between SIH and STH

There are 16 articles that reported the long‐term survival of patients who underwent SIH or STH.^10^, ^11^, ^12^, ^13^, ^15^, ^16^, ^17^, ^18^, ^19^, ^21^, ^22^, ^23^, ^24^, ^25^, ^26^, ^32^ Table 2 shows the 3‐year, 5‐year OS and the corresponding mortality rates. In the 13 articles reporting 3‐year OS, the 3‐year OS rates range of SIH and STH were 51.3%–74.5% and 10%–85.0%, respectively, and the corresponding 3‐year mortality rates were 816/2012 (40.6%) and 751/2176 (34.5%), respectively. In the 14 articles reporting 5‐year OS, the 5‐year OS rates range of SIH and STH were 14.0%–56.0% and 0–76.5%, respectively, and the corresponding 5‐year mortality rates were 1255/2025 (70.0%) and 1137/2150 (52.9%). The results of meta‐analysis showed that SIH did not increase the risk of postoperative 3‐year or 5‐year mortality for patients with SCLM, and the pooled OR was 1.090 (95% CI: 0.903–1.316; *p* = .368) and 1.077 (95% CI: 0.926–1.253; *p* = .337) respectively for SIH compared with STH. There was moderate heterogeneity in this analysis (*p* = .062; *I* ^2^ = 40.8%; *p* = .107; *I* ^2^ = 33.5%) (Figure 2C,D), which might come from one study that had a large difference with other studies, as shown by sensitivity analysis.^12^ (Figure S1C,D).

## 4 DISCUSSION

Colorectal cancer is a common clinical malignant tumor, and liver is the most common distant metastasis organ. About 15%–25% of colorectal cancers are accompanied with SCLM.^2^, ^3^, ^4^, ^5^ In 1980s, surgery was not a treatment option for SCLM patients, and the life expectancy of these patients was only 6–12 months.^33^, ^34^ For these patients, systemic chemotherapy or interventional therapy were attempted to improve the prognosis, but the effect was poor.^35^ Thus, some surgeons began to try to implement surgery for the SCLM patients. Although the incidence of postoperative complications was high at that time, some patients still achieved satisfactory long‐term prognosis.^33^, ^36^ Gradually, SCLM were no longer the absolute taboo of surgery.

However, the timing of surgery for SCLM patients has always been under clinical debate since it was proposed that surgery was suitable for these patients.^37^ For more than 40 years, whether SIH or STH should be performed for SCLM patients has always been controversy.^11^, ^12^, ^13^, ^14^, ^15^, ^16^, ^17^, ^18^, ^19^ Previous articles reported that SIH increased the risk of surgery, leading to higher incidence of surgical complications or perioperative mortality.^38^ However, thanks to the improvement of preoperative imaging, anesthesia and intensive care (28), and the improvement of surgical techniques, especially the progress of hepatectomy technology, the risk of postoperative complications and perioperative mortality have significantly reduced in recent years.^39^ Some studies suggested that SIH could combine colorectal cancer surgery and liver metastasis surgery into one operation, thus reducing the number of operations, and preventing postoperative immunosuppression and tumor growth caused by repeated operations.^40^ In addition, SIH can avoid the delayed treatment of liver metastases during STH, and early resection of liver metastases can improve the prognosis.^41^ However, some studies suggested that STH could contribute to detect the subclinical liver metastases and more underwent thorough hepatectomy.^24^ In addition, the pathology of lymph node metastasis can be obtained before hepatectomy for patients underwent STH, which can contribute to the choice of treatment options.^42^

Therefore, to resolve the controversy between SIH and STH, especially for SCLM patients under SIMH and STMH, we selected newly published articles after 2010, and conducted a meta‐analysis to compare the effect of SIH and STH, SIMH and STMH on postoperative complications, perioperative mortality and long‐term prognosis. Our analysis showed that SIH did not increase the risks of postoperative complications, perioperative mortality, 3‐year mortality or 5‐year mortality. In other words, SIH had no significantly difference compared with STH in postoperative complications, perioperative mortality and long‐term prognosis for SCLM patients.

The early studies generally believed that the occurrence of postoperative complications and perioperative mortality were related to the extent of hepatectomy. And it has been reported that major hepatectomy would increase the risk of postoperative complications and perioperative mortality for SCLM patients.^40^, ^43^ However, our meta‐analysis showed that SIMH did not increase the risk of postoperative complications or perioperative mortality as compared with STMH for SCLM patients. Further analysis showed that, the proportion of major hepatectomy in SIH or STH was 509/1979 (25.7%) and 1281/2390 (53.6%), respectively, as reported by 13 out of the 18 articles included in this study. The proportion of major hepatectomy in SIH was significantly lower than that in STH, indicating that most surgeons were still relatively cautious about the application of major hepatectomy in SIH, and only the patients who have been fully evaluated and screened were considered for receiving major hepatectomy in SIH.

In this study, we found that the literature published by Bogach et al. had a large heterogeneity compared with other articles, and the possible reasons might be: 1. There were 1166/2738 (43%) SCLM patients, which was higher than the proportion reported in previous literature (15%–25%).^2^, ^3^, ^4^, ^5^ 2. In this literature, the proportion of elderly patients underwent SIH was relatively higher than other articles. There were 47.5% of the patients underwent SIH with ages over 70 years old, while there were only 24.5% of the patients underwent STH. The tolerance of elderly patients for surgery is relatively poor. Some articles clearly suggested that patients over 70 years old should avoid SIH.^44^ Therefore, the differences in age distribution between SIH and STH may be other reason for the heterogeneity in this study.

There are two limitations in this study. Firstly, there is a lack of sufficient data on long‐term prognosis of major hepatectomy. Thus, more data is needed to analyze the differences in long‐term prognosis between SIMH and STMH. In addition, due to the limitations of the included articles, we cannot well distinguish the differences in preoperative and postoperative chemotherapy between SIH and STH. Fortunately, our sensitivity analysis showed that the heterogeneity among include literatures is small. Therefore, we have reason to believe that there is little difference in preoperative or postoperative chemotherapy among include literatures. Of course, our research needs further prospective researches to confirm.

## 5 CONCLUSION

There is no significant difference in postoperative complications, perioperative mortality and long‐term prognosis between SIH and STH for SCLM patients. Furthermore, postoperative complications and perioperative mortality also had no significant difference between SIMH and STMH. According to recent articles, the proportion of major hepatectomy in SIH was much lower than that in STH. Therefore, for patients who need major hepatectomy, STH is still the main method, and some patients may be considered for SIH after a comprehensive assessment of the patient's age, physical condition and other factors.

## AUTHOR CONTRIBUTIONS

**Jianwei Liu:** Data curation (lead); formal analysis (lead); writing – original draft (lead); writing – review and editing (lead). **Yong Xia:** Data curation (equal); formal analysis (equal); writing – original draft (lead); writing – review and editing (lead). **Xiaorong Pan:** Data curation (equal); formal analysis (equal); writing – original draft (lead); writing – review and editing (lead). **Zhenlin Yan:** Data curation (equal); formal analysis (supporting). **Lei Zhang:** Data curation (equal); formal analysis (supporting). **Zhao Yang:** Data curation (equal); formal analysis (supporting). **Yeye Wu:** Data curation (equal); formal analysis (supporting). **Hui Xue:** Data curation (equal); formal analysis (supporting). **Shilei Bai:** Data curation (equal); formal analysis (supporting). **Feng Shen:** study concept (lead), design (lead), Data curation (lead); formal analysis (lead); writing – original draft (lead); writing – review and editing (lead). **Kui Wang:** study concept (lead), design (lead), Data curation (lead); formal analysis (lead); writing – original draft (lead); writing – review and editing (lead).

## CONFLICT OF INTEREST

The authors have stated explicitly that there are no conflicts of interest in connection with this article.

## ETHICAL APPROVAL

Not applicable.

## ETHICS COMMITTEE APPROVAL

Ethics committee approval not received for this study as there are no human or animal subjects directly recruited.

## CONSENT FOR PUBLICATION

Not applicable.

## INFORMED CONSENT

None.

## Supporting information


**Figure S1:** The sensitivity analysis of SIH on surgical safety, long‐term prognosis compared with STH. (A) The sensitivity analysis of all articles reported postoperative complications; (B) The sensitivity analysis of all articles reported perioperative mortality; (C) The sensitivity analysis of all articles reported postoperative 3‐year mortality; (D) The sensitivity analysis of all articles reported postoperative 5‐year mortality.

**Table S1:** Quality of literatures included in this systematic review and meta‐analysis.

## Data Availability

Data sharing is not applicable to this article as no new data were created or analyzed in this study.
