# Supplementary figures and images for: Simultaneous versus staged major hepatectomy (≥3 liver segments) for outcomes of synchronous colorectal liver metastases: A systematic review and meta‐analysis
Source: Cancer Rep (Hoboken). 2022 Jun 26;5(8):e1617. doi: 10.1002/cnr2.1617 (PMC9351651; doi:10.1002/cnr2.1617)

Supplement Figure 1

A

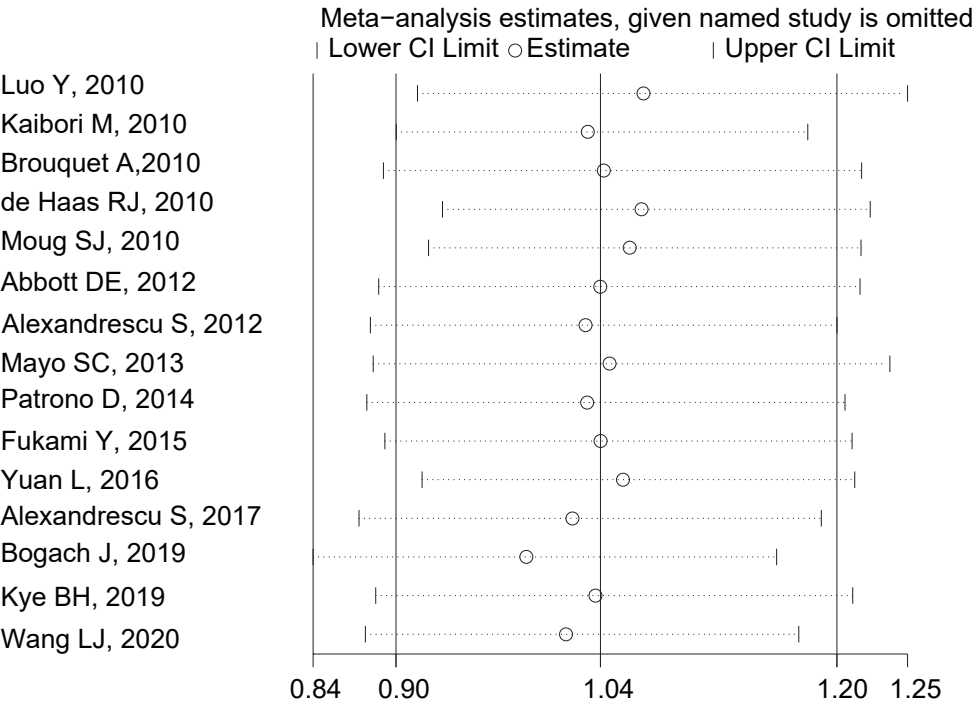

B

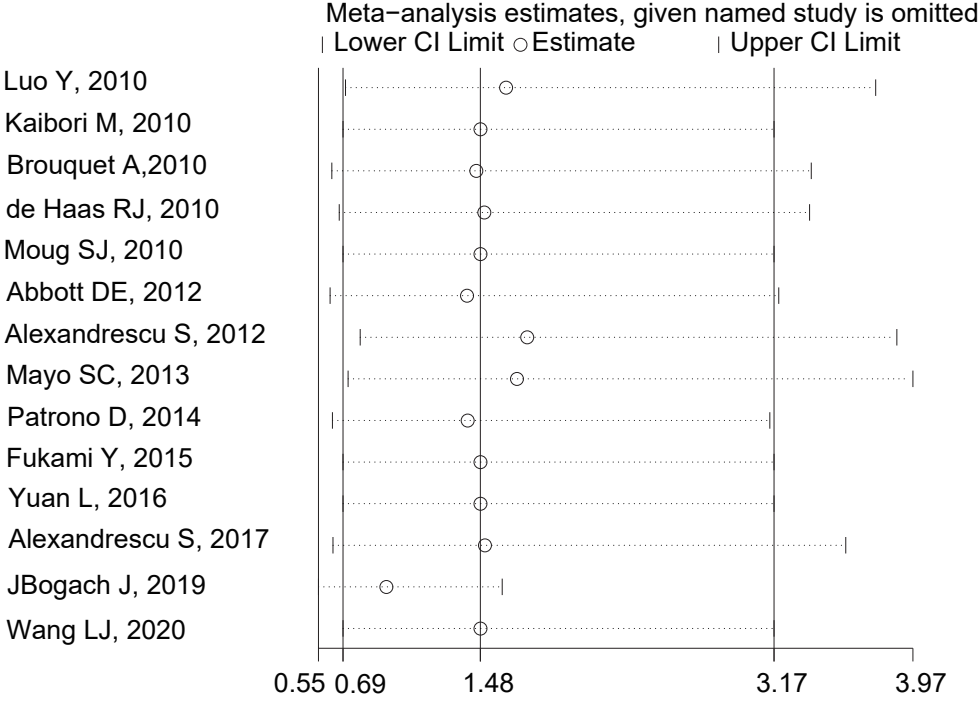

C

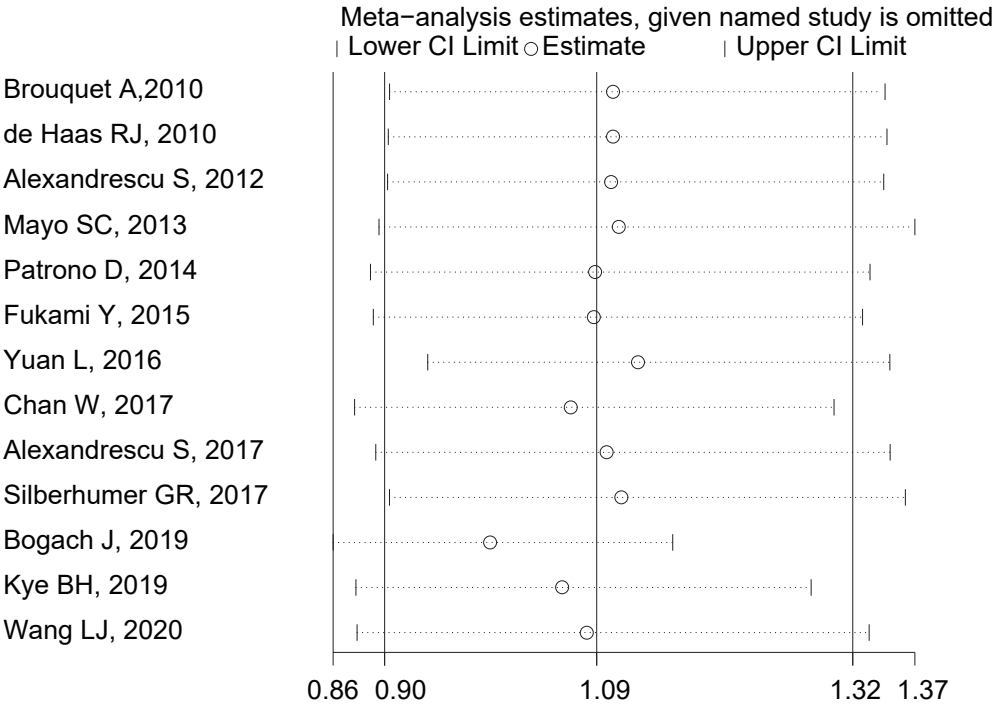

D

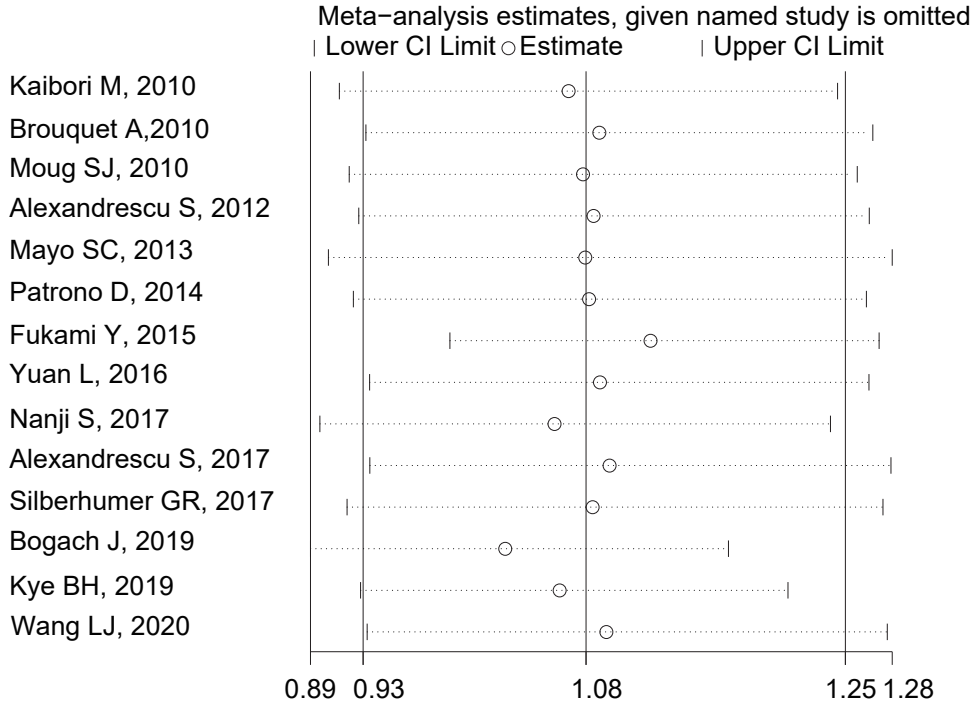

Supplement: Supplementary file 1 — Figure S1: The sensitivity analysis of SIH on surgical safety, long‐term prognosis compared with STH. (A) The sensitivity analysis of all articles reported postoperative complications; (B) The sensitivity analysis of all articles reported perioperative mortality; (C) The sensitivity analysis of all articles reported postoperative 3‐year mortality; (D) The sensitivity analysis of all articles reported postoperative 5‐year mortality. [file CNR2-5-e1617-s002.pdf]
