# Supplementary material for: Simultaneous versus staged major hepatectomy (≥3 liver segments) for outcomes of synchronous colorectal liver metastases: A systematic review and meta‐analysis
Source: Cancer Rep (Hoboken). 2022 Jun 26;5(8):e1617. doi: 10.1002/cnr2.1617 (PMC9351651; doi:10.1002/cnr2.1617)
Supplement: Supplementary file 2 — Table S1: Quality of literatures included in this systematic review and meta‐analysis. [file CNR2-5-e1617-s001.docx]

| **Supplement table 1. Quality of Literatures Included in This Systematic Review and Meta-Analysis** | | | | | | | | |
| --- | --- | --- | --- | --- | --- | --- | --- | --- |
| **Study** | **Participation** | **Study attrition** | **Prognostic factor measurement** | **Outcome measurement** | **Study confounding** | **Statistical analysis and reporting** | **Final score used for analyses** | **Ref** |
| [Luo Y,](https://www.ncbi.nlm.nih.gov/pubmed/?term=Luo%20Y%5BAuthor%5D&cauthor=true&cauthor_uid=20676791) 2010 | low | moderate | high | low | moderate | low | 10 | 25 |
| [Kaibori M](https://www.ncbi.nlm.nih.gov/pubmed/?term=Kaibori%20M%5BAuthor%5D&cauthor=true&cauthor_uid=20112062),2010 | low | moderate | low | low | moderate | low | 8 | 17 |
| [Brouquet A](https://www.ncbi.nlm.nih.gov/pubmed/?term=Brouquet%20A%5BAuthor%5D&cauthor=true&cauthor_uid=20510802),2010 | low | low | moderate | high | moderate | high | 12 | 10 |
| [de Haas RJ,](https://www.ncbi.nlm.nih.gov/pubmed/?term=de%20Haas%20RJ%5BAuthor%5D&cauthor=true&cauthor_uid=20578183) 2010 | low | low | low | low | low | low | 6 | 16 |
| Moug SJ, 2010 | moderate | moderate | moderate | low | moderate | moderate | 11 | 27 |
| [Abbott DE,](https://www.ncbi.nlm.nih.gov/pubmed/?term=Abbott%20DE%5BAuthor%5D&cauthor=true&cauthor_uid=22560316) 2012 | low | moderate | moderate | low | moderate | moderate | 10 | 14 |
| [Alexandrescu S,](https://www.ncbi.nlm.nih.gov/pubmed/?term=Alexandrescu%20S%5BAuthor%5D&cauthor=true&cauthor_uid=22844827) 2012 | low | low | moderate | low | low | low | 7 | 28 |
| Mayo SC, 2013 | moderate | high | moderate | moderate | moderate | low | 13 | 15 |
| [Patrono D,](https://www.ncbi.nlm.nih.gov/pubmed/?term=Patrono%20D%5BAuthor%5D&cauthor=true&cauthor_uid=24841686) 2014 | low | low | high | low | low | low | 8 | 13 |
| [Fukami Y,](https://www.ncbi.nlm.nih.gov/pubmed/?term=Fukami%20Y%5BAuthor%5D&cauthor=true&cauthor_uid=26007322) 2015 | low | low | moderate | low | low | moderate | 8 | 26 |
| Yuan L, 2016 | low | low | low | low | low | low | 6 | 19 |
| Chan W, 2017 | low | low | moderate | low | low | low | 7 | 37 |
| Nanji S, 2017 | low | low | low | low | low | low | 6 | 29 |
| [Alexandrescu S,](https://www.ncbi.nlm.nih.gov/pubmed/?term=Alexandrescu%20S%5BAuthor%5D&cauthor=true&cauthor_uid=28675363) 2017 | low | low | high | low | low | low | 8 | 30 |
| [Silberhumer GR,](https://www.ncbi.nlm.nih.gov/pubmed/?term=Silberhumer%20GR%5BAuthor%5D&cauthor=true&cauthor_uid=27079362) 2017 | low | moderate | moderate | low | low | low | 8 | 31 |
| Bogach J, 2019 | high | low | low | low | moderate | low | 9 | 12 |
| Kye BH, 2019 | low | low | low | low | low | low | 6 | 18 |
| [Wang LJ,](https://www.ncbi.nlm.nih.gov/pubmed/?term=Wang%20LJ%5BAuthor%5D&cauthor=true&cauthor_uid=31952490) 2020 | low | low | low | moderate | moderate | moderate | 9 | 11 |

Abbreviation: Ref = References
